# Management of Deep Infection after Instrumentation on Lumbar Spinal Surgery in a Single Institution

**DOI:** 10.1155/2015/842010

**Published:** 2015-07-26

**Authors:** Jung-Tung Liu, Wen-Jui Liao, Cheng-Siu Chang, Yung-Hsiang Chen

**Affiliations:** ^1^School of Medicine, College of Medicine, Chung Shan Medical University, Taichung 40201, Taiwan; ^2^Departments of Neurosurgery, Nuclear Medicine, and Radiology, Chung Shan Medical University Hospital, Taichung 40201, Taiwan; ^3^Graduate Institute of Integrated Medicine, College of Chinese Medicine, China Medical University, Taichung 40402, Taiwan; ^4^Department of Medical Research, China Medical University Hospital, Taichung 40402, Taiwan; ^5^Department of Psychology, College of Medical and Health Science, Asia University, Taichung 41354, Taiwan

## Abstract

Postoperative surgical site infections (SSIs) are more common complications after spinal surgery. SSIs often require extended hospitalisation and may worsen overall clinical outcomes. A retrospective database review of consecutive patients with traditional open lumbar spinal surgery was performed. SSIs patients were identified and reviewed for clinically relevant details, and postoperative SSIs' incidence was calculated for the entire cohort as well as for subgroups with or without spinal implants. In 15 years, 1,176 patients underwent open lumbar spinal surgery with spinal implants and 699 without. Thirty-eight developed postoperative SSIs. Total SSI rate for the entire group was 2.03%. The incidence of postoperative SSIs in the nonimplant group was relatively low. Patients received antibiotics, hyperbaric oxygen therapy, and wet dressing. We provided the precise rates of postoperative SSIs in traditional open spinal surgery obtained from a single-centre data. Patients with spinal implants had higher SSIs' incidence than those without.

## 1. Introduction

Postoperative surgical site infection (SSI) is one of the most common complications [1–3] after spinal surgery [4, 5]. The incidence of spinal SSIs reported in the literature is 0.7%–16.0% [6]. These infections often require extended antibiotic therapy, repeated surgery for wound debridement, hardware removal, and prolonged hospitalisation [7]. It dramatically increases utilisation of healthcare resources and worsens overall clinical outcomes [8, 9].

Several complicated procedures result in higher infection rates. Therefore, spinal surgeries carry a higher risk of infection compared with other orthopaedic procedures [10]. Another problem of the increasing complexity of spinal surgeries is increased operation time, which is a well-known intraoperative risk factor for SSIs [11–13]. Besides surgical factors, patient's preoperative characteristics (increased age and body mass index (BMI), smoking, diabetes, steroid use, malnutrition, and previous surgical infection) could also account for an increase in the number of postoperative complications [11–13].

The management of SSIs has become increasingly important. Considering the complexity of spinal procedures, preventive interventions have the potential to improve a patient's overall outcome [14, 15]. Furthermore, these interventions may decrease the duration of hospital stay and postoperative recovery time, thereby lowering medical expenses.

## 2. Patients and Methods

### 2.1. Ethics Statement

The approval of local ethics committee for this research was permitted. The protocol conforms to the Declaration of Helsinki and the Institutional Review Board of Chung Shan Medical University (Taichung, Taiwan) approved the study by expedited review (Approval Reference number: CS15026).

### 2.2. Retrospective Database Review

We performed a retrospective review of prospectively collected databases of consecutive patients who underwent open lumbar spinal surgery between February 1998 and December 2012 by experienced surgeons at our hospital. During this 15-year period, 1,176 lumbar spinal surgeries were performed, and the procedures included the following: simple decompression procedures such as micro- or endoscopic discectomy or foraminotomy or decompression of stenosis, arthrodeses (e.g., posterolateral interbody, posterior/transforaminal interbody, and lateral interbody), filum detethering, or syrinx shunting. All procedures were performed using a standard surgical scrub and preparation and draping of patients after administering general anaesthesia were carried out. All patients received a single dose of intravenous antibiotics immediately before surgery (1-2 g of cefazolin or 1 g of vancomycin in those reporting an allergy to penicillin or cephalosporin). This regimen was repeated as required during surgeries lasting > 4 h.

The databases included documentation of all perioperative complications. Identification of SSIs as classified according to the criteria set by the Centres for Disease Control and Prevention was studied. An infection was considered to be SSI if it occurred at the site of the surgery within 30 days postoperatively or within 1 year if the procedure included placement of a foreign body (e.g., an implant). Cases with SSI were identified and confirmed through microbiological cultures. The incidence of postoperative SSIs was calculated for the entire cohort as well as for subgroups with or without spinal implants. Positive cases of SSI were reviewed for clinically relevant details.

Demographic and preoperative variables were collected from medical records using a standardised data collection form by an investigator who was not involved in the initial treatment. Information regarding preoperative risk factors was derived from standardised and routinely recorded data as reported in the patient charts. Surgical-level risk factors that could be considered possible risk factors for infection were derived from surgical reports of the surgeons' database. When the data collection was completed, all data were checked by a second investigator.

Preoperative patient-level risk factors that were reviewed included age at the time of surgery, sex, height, weight, and diagnosis. Additionally, smoking habits, comorbidity, and previous lumbar surgeries were recorded, and BMI was calculated. Registered type of comorbidity included diabetes, rheumatoid arthritis, and cardiovascular and pulmonary diseases.

### 2.3. Statistical Analysis

Two-tailed independent* t*-tests, Chi-square tests, or appropriate nonparametric alternatives were used to identify differences between the groups of patients with or without implants. Probability values of < 0.05 were considered statistically significant. All data were analysed using SigmaPlot statistical software (SAS Institute, San Jose, CA, USA).

## 3. Results

### 3.1. Deep Infection after Instrumentation on Lumbar Spinal Surgery

Over the past 15 years, 1,875 open lumbar spinal surgeries (1,176 patients with spinal implants and 699 patients without spinal implants) were performed at our medical centre. Thirty-eight postoperative SSIs were detected. The total SSI rate for the entire group was 2.03% (31 (2.64%) in the implant group and 7 (1.00%) in the nonimplant group). The incidence of wound infection and operation time in the nonimplant group was relatively low (*P* < 0.05). No significant differences in age, sex, extent of bleeding, amount of irrigation, indwelling of drainage tube, days of the initial onset of infection, and duration of hospital stay were ascertained for both groups (Table 1).

### 3.2. Underlying Disease, Infection Germ, Treatments, and Outcome

Approximately 50% patients with postoperative infection have comorbidity associated with diabetes and hypertension. There was no significant difference between any type of underlying diseases and BMI between these two groups. The type of bacteria and sample cultures in different groups are listed in Table 2. The most common organism isolated from wound cultures was* Staphylococcus aureus*, followed by methicillin-resistant* S. aureus* (MRSA). Patients received antibiotics, hyperbaric oxygen (HBO) therapy, and wet dressing, and most showed good outcome. However, some patients developed low back pain and neuralgia after postoperative infection.

## 4. Discussion

Postoperational SSIs after spinal surgery remain a serious condition, leading to major complications and worse outcomes [6, 16]. Although the generalized adoption of preoperative antibiotic prophylaxis has served to decrease SSI rates to as much as 50%, it has not completely eliminated them [17–19]. Recently, a review of 2,316 patients who underwent a wide variety of open spinal surgeries over 5 years found an overall infection rate of approximately 2% [20]. Our data are consistent with those results. The most common pathogen isolated from wound cultures was* Staphylococcus* species (predominantly* S. aureus*) [6, 11, 21], followed by MRSA [22, 23], which was responsible for several difficult-to-treat infections [24]. Our database provided additional documentation of several factors that may be related to the occurrence of wound infection based on the administration of prophylactic antibiotics, duration of operation time, estimated blood loss, length of hospitalisation, or patient comorbidities. In addition, there was documentation of causative organisms on the management of infection or surgical outcomes.

Recently, another retrospective review also provided the rates of postoperative wound infection after a broad range of spinal procedures based on the cases predominantly performed by fellowship-trained spinal surgeons [25]. The large number of cases in that review enabled assessment of infection rates for relatively uncommon procedures, including those performed on paediatric patients. Their database also enabled stratification of cases and assessment of the corresponding infection rates based on surgical factors, including primary* versus* revision status, use of implants, and fusion approach, and whether minimally invasive techniques were used. The 2% total infection rate in this series was comparable with that in previously reported series, which included a diverse representation of spinal procedures, with the infection rate ranging from 0.9% to 4.4% [11, 20, 26, 27]. The rates in the present study, in general, were comparable with those in the previous reports.

The risk factors for infection included diabetes, elevated serum glucose levels, and inappropriate timing or dosing of preoperative antibiotics [6]. A case-control study identified incontinence, posterior surgical approaches, tumour resection procedures, and obesity as independent risk factors for postoperative SSIs [13]. The role of diabetes and high BMI as risk factors has been supported in other studies [28–31]. Information on preoperative risk factors for SSI, such as diabetes, previous surgery, obesity, previous exposure to radiation, and smoking, was not consistently recorded in our databases. Therefore, comparison of the group of patients in our study with those in other published cohorts based on these variables was not possible. More formal case-control or randomized studies on this topic could better answer these important questions.

HBO has been reported to heal postoperative spinal infections in adults with intact osteosynthesis material [32]. The therapeutic effect of HBO treatment with regard to infections is mainly attributable to reduction of hypoxia in tissues with significant improvement in leucocyte phagocytic killing capacity [33]. Larsson et al. have evaluated possible benefits of HBO therapy in the treatment of deep postoperative infections in patients with neuromuscular spine deformity, suggesting that HBO therapy is a safe and potentially useful adjuvant treatment to the standard therapy of early postoperative deep infections [34, 35].

The present study also cannot answer the possible reasons for lower infection rates after open lumbar spinal surgery. However, several potential mechanisms may have important roles [6]. To avoid SSIs after spinal surgery, ultimately, there is no replacement for sterile methods, meticulous hemostasis and closure, and appropriate preoperative antibiotic prophylaxis. Further study is required to validate these findings through more direct comparisons of traditional open versus minimally invasive approaches.

Based on our data and those from previous studies [25], the rates of postoperative wound infections were significantly higher for cases that included fusion or implants than those that did not. It is important to recognize that these data do not necessarily suggest a causative link between infection and performance of fusion or implantation but rather likely reflect a greater complexity and associated risk for cases that require fusion or implantation. The overall infection rate for procedures performed with a minimally invasive approach was significantly lower compared with that for those without. Importantly, several procedures that are commonly performed using a minimally invasive approach (e.g., lumbar discectomy), in general, have a relatively low infection rates, whereas more complex procedures that are typically conducted using a traditional open approach (e.g., degenerative scoliosis or neuromuscular kyphosis) have substantially higher infection rates [28].

## 5. Conclusions

We detected a low rate of SSIs in this large series of patients who underwent open lumbar spinal surgery. In addition, we provided the precise rates (2.03%) of postoperative wound infections in traditional open spinal surgery from a single-center data. The incidence of SSIs in patients with spinal implants was higher than that in those patients without spinal implants. Further large-scale prospective studies using a clear definition of complication are necessary to ascertain the true incidence of postoperative complications in spinal surgery. Our data provide a general benchmark of infection rates as a basis for ongoing efforts to improve safety of care.

## Figures and Tables

**Table 1:** Demographic data of patients with postoperative infection.

	Implant group	Nonimplant group	*P* value
Postoperative infection [*n*/total (%)]	31/1176 (2.64)	7/699 (1.00)	<0.05
Age (years, mean ± SD)	65.5 ± 12.9	67.6 ± 11.8	0.702
Gender, male/female	16/15	2/5	0.410
Operation time (hours)	3.4 ± 0.8	2.7 ± 0.8	<0.05
Bleeding amount (mL)	826.5 ± 361.7	564.3 ± 319.2	0.086
Irrigation amount (mL)	2080.6 ± 817.5	1464.3 ± 625.4	0.071
Drainage tube indwelling, Y/N	30/1	6/1	0.339
Days for infection start	5.2 ± 0.6	5.0 ± 0.0	0.344
Hospital stay (days)	32.6 ± 3.4	33.7 ± 7.7	0.695

**Table 2:** Underlying diseases, infection germ, treatments, and outcome in patients with postoperative infection.

	Implant group	Nonimplant group
Underlying diseases		
Diabetes	15	4
Hypertension	15	3
Osteoporosis	9	1
Poor nutrition	2	1
Traumatic injury	2	0
Coronary artery disease	2	0
Uremia	2	0
Rheumatoid arthritis	1	1
High BMI	1	0
Low BMI	1	0
Cancer	0	2
Hyperthyroidism	0	1
Nil	4	0
Infection germ		
*Staphylococcus *	17	2
MRSA	9	3
ORSA	0	2
*E. coli *	2	0
*Pseudomonas *	2	0
*Proteus *	1	0
Treatments		
Antibiotics	31	7
Hyperbaric oxygen	31	6
Wet dressing	31	7
Nutrition supply	1	1
Outcome		
Good	20	6
Low back pain	9	0
Neuralgia	1	0
Nutrition supply	1	0
Expiry (sepsis)	0	1
